# Dr. James Granger

**Published:** 1914-02

**Authors:** 


Dr. James Granger, (not listed in State Directory) died at his home in Mayville, December 21, 1913, aged 72.

